# Clear Cell Acanthoma: A Review of Clinical and Histologic Variants

**DOI:** 10.3390/dermatopathology7020005

**Published:** 2020-08-25

**Authors:** Arif Usmani, Syeda Qasim

**Affiliations:** 1 Benchmark Diagnostics, Middleburg Heights, OH 44130, USA; 2 School of Graduate Studies, Rutgers University, New Brunswick, NJ 08901, USA

**Keywords:** clear cell acanthoma, trichilemmoma, glycogen accumulation, Cowden syndrome

## Abstract

Degos and Civatte first described clear cell acanthoma (CCA) in 1962 and later in a review article found that, in most instances, the lesion was a solitary red-brown dome-shaped papule that involved the distal lower extremity. The first morphologic variant of CCA was reported as a “giant form of the acanthoma of Degos” which measured 45 × 40 mm, about twice the size of the largest CCA documented earlier. Since then, many variants of CCA have been described, including polypoid, pigmented and atypical. Herein, we describe a new variant of CCA and add another example of the polypoid variant to the literature. The new variant exhibits cellular features of trichilemmoma but architecturally differs from it. We also attempt to broaden the list of CCA variants summarized by Tempark and Shwayder by adding ours and a few more examples of CCA. The new variants of CCA include verrucous, linear, subungual and trichilemmal.

## 1. Introduction

Degos and Civatte first described clear cell acanthoma (CCA) in 1962 [1]. Later in 1970, they summarized the experience of the past eight years in another article [2]. They noted that CCA was not uncommon, as more than 100 cases were added to the literature during that period. They also noted that retrospective diagnosis was made by several authors who had put aside similar cases to be studied at leisure [3,4,5].

During their review [2], Degos and Civatte found that, in most instances, the lesion was solitary and involved the distal lower extremity. Most of the lesions occurred between the fifth and seventh decade and had no gender proclivity. The size of the lesion ranged from 5 to 20 mm in diameter. However, one lesion, reported by Duperrat et al. [6], measured 45 × 40 mm. Their case was the first description of a morphologic variant of CCA, the so-called “giant form of the acanthoma of Degos” (“forme geante de l’Acanthome de Degos”). Since then, many variants of CCA have been described, including polypoid, pigmented and atypical. In this paper, we describe a new variant of CCA and add another example of the rare polypoid variant to the literature. We also attempt to broaden the list of CCA variants summarized by Tempark and Shwayder [7] by adding ours and a few more examples of CCA, that have been reported before and subsequently, to their review.

The exact etiology of clear cell acanthoma is unknown as it is unclear whether clear cell acanthoma is a neoplastic process or an inflammatory dermatosis [8]. Although CCA presents most often as a solitary lesion, it bears significant similarities to psoriasis in terms of histopathology, cytochemistry, immunohistology and even on dermoscopy. Moreover, the EMA positivity noted in CCA is also seen in reactive, inflammatory conditions. The cytokine expression of CCA is also similar to that seen in other forms of inflammatory dermatoses [9]. These findings suggest that CCA is probably a reactive process rather than a neoplastic one. 

## 2. Material and Methods

### 2.1. Case 1

**Figure 1:**
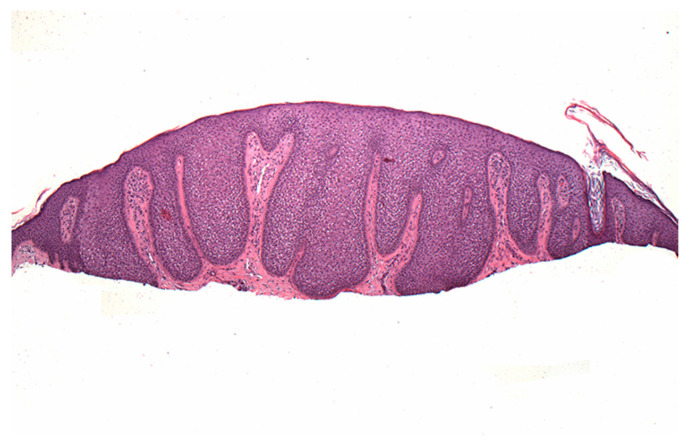
A dome-shaped lesion exhibits pale keratinocytes with a large perinuclear vacuole reminiscent of trichilemmal differentiation.

**Figure 2:**
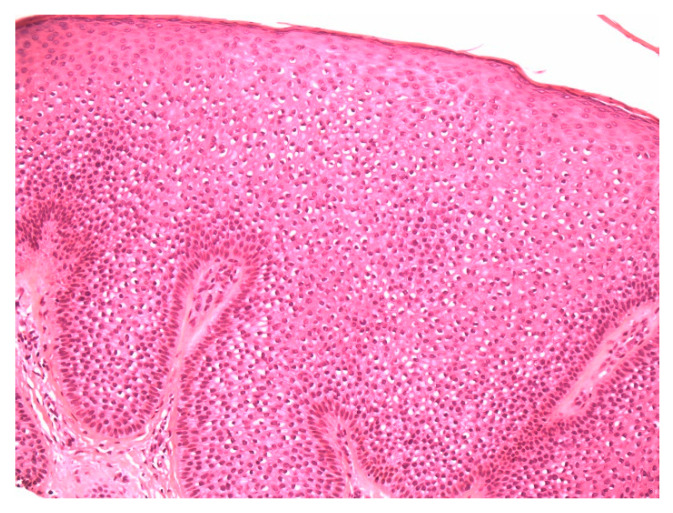
High-power view demonstrating distinct large perinuclear vacuoles and peripheral palisading of basal keratinocytes.

**Figure 3:**
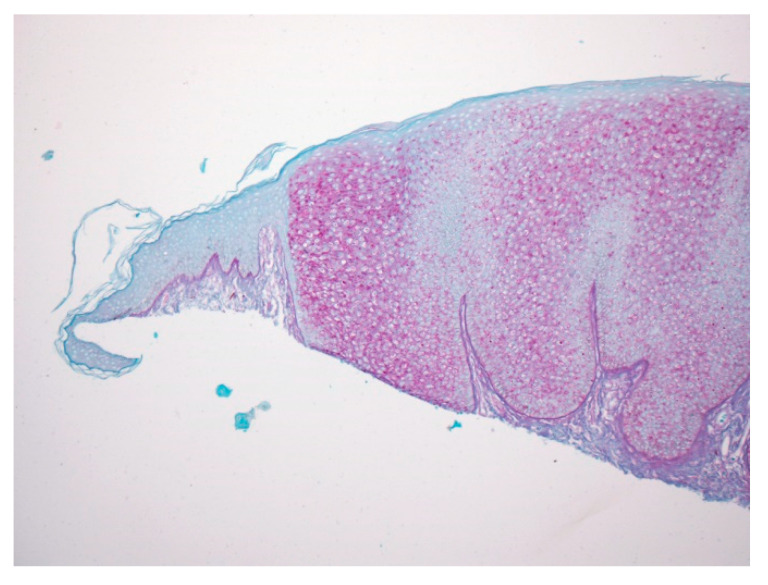
With PAS staining, the lesion is sharply demarcated from the normal epidermis, suggesting glycogen accumulation in lesional keratinocytes.

**Figure 4:**
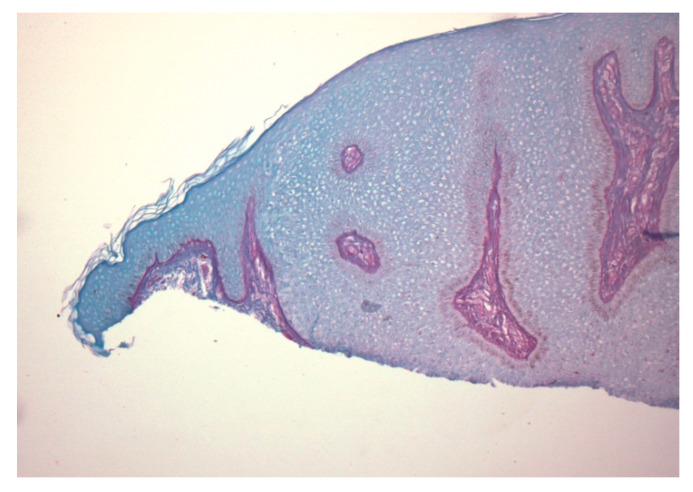
The cytoplasm highlighted by the PAS stain is negative after diastase treatment, confirming glycogen accumulation.

**Figure 5:**
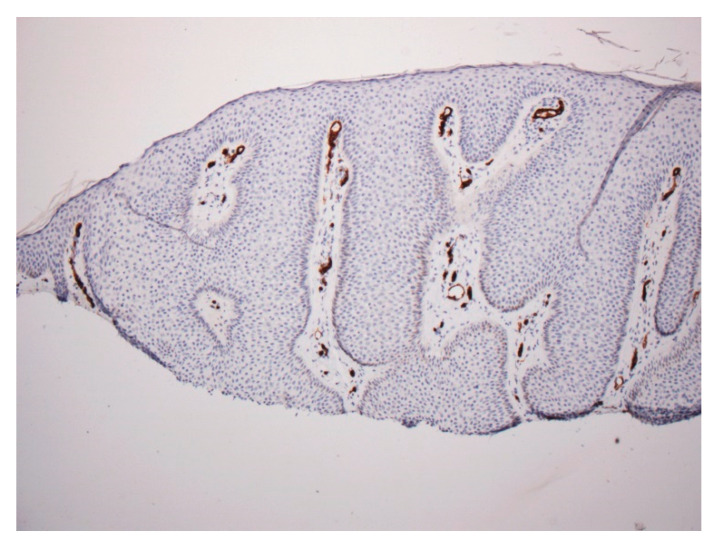
No keratinocyte staining is noted with CD34 stain.

A 74-year-old female presented with a 3 mm pink papule on the right side of the neck with a clinical impression of basal cell carcinoma. The patient was being treated for anxiety and depression and hypercholesterolemia. There was no history of Cowden syndrome. A 4 × 3 mm shave specimen was processed and revealed circumscribed acanthosis of the epidermis. Keratinocytes showed typical pale cytoplasm and large perinuclear vacuoles reminiscent of trichilemmal keratinocytes (Figure 1). Large perinuclear vacuoles and peripheral palisading of basal keratinocytes were distinctly seen in high-power view (Figure 2). The involved epidermis showed a sharp demarcation from the normal epidermis which was accentuated with PAS staining (Figure 3). The stratum corneum showed focal parakeratosis without prominent neutrophils. The papillary dermis was elongated with the thin overlying suprapapillary epidermis. PAS stain highlighted keratinocyte cytoplasm which was negative after diastase treatment, suggesting glycogen accumulation (Figure 4). Keratinocytes were negative with CD34 stain (Figure 5).

### 2.2. Case 2

**Figure 6:**
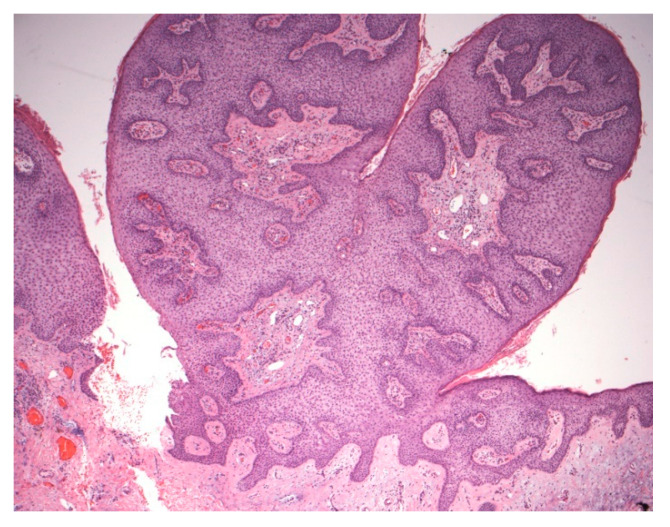
Polypoid lesion with prominent acanthosis and keratinocytes containing pale cytoplasm. No peripheral palisading or perinuclear vacuoles are noted in this variant.

A 69-year-old female presented with a polypoid lesion on the right of the neck with a clinical impression of irritated intradermal nevus. There was no other relevant history except that she was on Lipitor. A 9 × 6 mm shave specimen was processed and revealed a lesion with polypoid architecture, showing prominent acanthosis with keratinocytes containing pale cytoplasm (Figure 6). No clear perinuclear vacuoles were identified. Neutrophils were noted in the parakeratotic stratum corneum and upper stratum malpighii. The papillary dermis was elongated with prominent vessels and lymphocytic infiltrate. Sharp demarcation from adjacent normal epidermis was noted. PAS stain highlighted keratinocyte cytoplasm which was negative after diastase treatment.

## 3. Discussion

**Figure 7:**
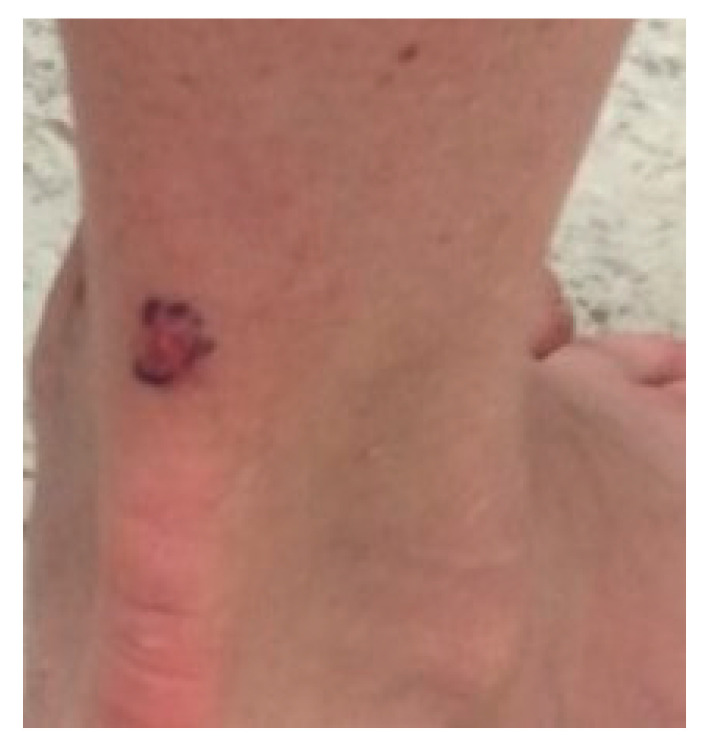
Clinical picture of classical CCA. Image courtesy: Karen Turgeon, MD.

The typical presentation of clear cell acanthoma is a red to brown dome-shaped papule on the leg of an elderly individual. (Figure 7) It is usually asymptomatic, but itching and bleeding following minor trauma are occasionally reported. Scaling and moistening of the surface may be noted in some cases. Despite their consistent presentation, these lesions are frequently not recognized clinically and often biopsied to rule out cutaneous malignancy. Any variation in the presentation of CCA probably makes it more difficult to be recognized correctly.

Several variations in the clinical and histological appearance of CCA have been reported. Most recently, Tempark and Shwayder [7] have attempted to collect and list these variants of CCA in an article. Interestingly, their accompanying case report is another unique presentation of CCA in a 4-year-old African American boy which they did not include in their list. This lesion was a 47 × 7 mm linear hypopigmented plaque on the left flank composed of coalescing papules which clinically resembled epidermal nevus and lichen striatus and appeared to be following Blaschko’s lines. Not counting this case, they described six main clinical and histologic variants of CCA, namely, giant, polypoid, pigmented, eruptive, atypical and cystic.

We recently came across two variants of CCA in our practice. One with novel trichilemmal differentiation and the other with polypoid architecture. These encounters prompted a review of the literature for variants of CCA. We found that the polypoid variant has already been described, but there was no mention of a trichilemmal variant. We add our case and a few more variants of CCA from the literature to expand the list of Tempark and Shwayder. Table 1 lists these and previous variants in the temporal order of their documentation. We will discuss each of these variants in detail.

### 3.1. Variants of CCA

#### 3.1.1. Giant CCA

The giant variety was the first deviation from the conventional form of CCA, reported in 1966 by Duperrat et al. [6]. This lesion was twice the size of the largest lesion described until that point and measured 45 × 40 mm. Since then, the “giant” variant has been arbitrarily considered to be any CCA greater than 30 to 40 mm regardless of its morphologic appearance. The largest lesion was described by Grossin et al. [10], which measured around 70 mm. The giant form can be solitary or multiple [11,12] and can be exophytic [13], plaque-like [14], polypoid [15] and cerebriform [16]. The case of Arida et al. [15] also showed a central endophytic component resembling keratoacanthoma, however, there was no significant cytologic atypia and therefore cannot be assigned to the atypical variant category.

#### 3.1.2. Mucosal CCA

Weitzner [17] described a CCA on the vermilion mucosa of the lower lip that was clinically thought to be leukoplakia. Two other cases from the lip area were reported earlier, but both were on the skin of the lip. There is only one other report of a mucosal variant, which was also pigmented [18]. However, a similar phenomenon, known as glycogen acanthosis (GA), has been described in the esophagus, mouth and upper respiratory tract [19,20]. Like the case of Weitzner, lesions on the tongue and larynx have also been reported to present as leukoplakia [19,20]. These lesions show pale glycogen-containing keratinocytes without the involvement of the basal layer, analogous to cutaneous CCA. In the esophagus, these lesions are incidental findings and are often mistaken for a plaque of candidiasis. In Cowden syndrome, extensive involvement of the esophagus with GA is noted. Interestingly, a recent article has documented the association of Cowden syndrome and multiple CCAs [21]. Turnbull et al. [22] also noted CCA in one of the two sisters they reported with Cowden syndrome.

#### 3.1.3. Verrucous CCA

There is only one report of the verrucous form of CCA in the Spanish literature [23]. This lesion occurred in a 72-year-old female with a history of repeated trauma. It is unclear whether the trauma was antecedent to the lesion.

#### 3.1.4. Polypoid CCA

**Figure 8:**
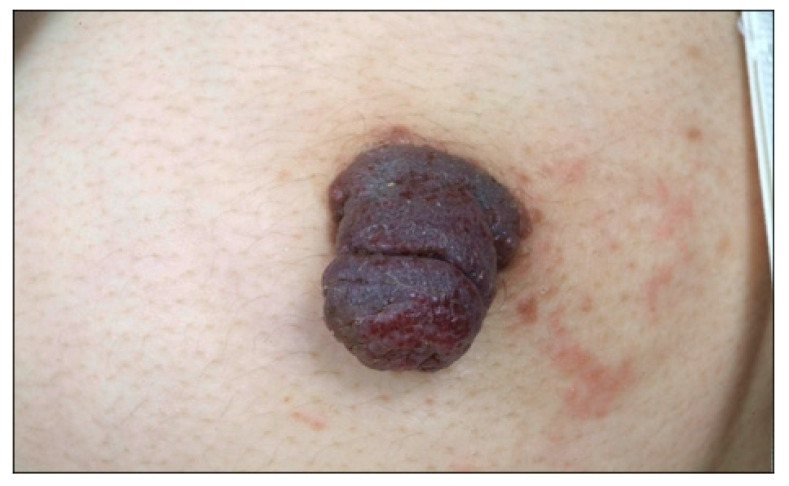
A solitary polypoid brown papule on the left nipple. Image courtesy: [26].

Several cases of polypoid, polypous or pedunculated CCA have been reported in various locations, including the head and neck, nipple, scrotum and lower extremity [24,25,26,27,28,29,30,31]. (Figure 8) We are reporting an additional case of polypoid CCA, which is also the second polypoid CCA reported on the neck. The first case on the neck was reported by Yang et al. [25], which was associated with a melanocytic nevus. A few reported cases of the giant variant were polypoid.

#### 3.1.5. Atypical CCA

Clear cell acanthoma is a benign neoplasm or a reactive condition depending on the etiologic postulation. However, both benign neoplastic and reactive conditions have the biologic potential to progress to dysplasia or cancer. Grunwald et al. [32] first reported two cases of CCA with cellular atypia and increased mitotic activity; although malignant degeneration was earlier suspected in two of over 100 cases reviewed by Degos and Civatte. Zak and Girerd [33] reported one, and Degos referenced the other unpublished case. In both these cases, some of the rete ridges showed complex downward proliferation into the dermis associated with some dyskeratosis in Degos’s case. Parsons and Ratz [34] reported squamous cell carcinoma in situ arising in CCA. Recently, Lin et al. [35] described a case similar to those of Grunwald’s but preferred to call it malignant clear cell acanthoma.

Shirai et al. [36] reported multiple CCA and multiple Bowen’s disease (BD) in a Japanese patient who had a questionable history of arsenic exposure. Although it was postulated by the authors that CCA may have progressed to BD due to the patient’s possible arsenic exposure, it appears that none of their CCAs had any atypical or dysplastic features. In their paper, they also referenced another case [37] from the Japanese literature, where CCA from the cheek showed bowenoid transformation.

The above-described cases appear to be incidental and do not warrant labeling CCA a premalignant condition.

#### 3.1.6. Pigmented CCA

The pigmented variant was first reported in the Spanish literature by Sanchez and Iglesias in 1975 [38], while Langer et al. described the first reported case in the English literature [39]. CCA is typically a hypomelanotic or amelanotic lesion due to a disturbance in melanin transfer from melanocytes to keratinocytes [39]. Hollmann and Civatte [40], upon electron microscopy, found intact melanocytes within CCA lacking in “melanin grains.” Later, in 1990, Fanti et al. [41] also confirmed the presence of melanocytes within CCA. Several other pigmented variants [42,43] have been reported, including one by Bugatti and Filosa [42] which showed hemosiderin in the dermis and called it “hemosiderotic clear cell acanthoma”. Pigmented CCA has also been reported on the lower lip mucosa [18] and on a finger which clinically resembled a melanocytic nevus [44]. Multiple pigmented CCAs have also been reported in an African patient [45].

It appears that, for CCA to be pigmented, melanocytic hyperplasia, with associated melanin-laden dendrites, is needed. Melanin transfer to keratinocytes is impeded possibly due to the latter being distended with glycogen, causing pressure blockage and resulting in the accumulation of melanin in melanocytic dendrites; however, the mechanism of melanin transfer from melanocytes to keratinocytes is more complex and the exact cause of impeded transfer in CCA is not known.

In contrast to healthy skin or pigmented seborrheic keratoses, pigmentation of the surface in pigmented CCA is not imparted by keratinocyte melanization but follows the pattern of melanocytic lesions.

#### 3.1.7. Eruptive CCA

As discussed, CCA can be solitary or multiple, however, in some instances, the number of lesions ranges from a few lesions to a few hundred lesions. In such cases, the term “eruptive” has been applied by the authors. Several cases of the eruptive variant have been reported [46,47,48,49,50]. The first of such cases was reported by Burg et al. [46] as eruptive hamartomatous CCA in a 38-year-old female with over 100 lesions on her legs, arms, and trunk. Innocenzi et al. [47] found approximately 400 papules of CCA in a 32-year-old female on the upper and lower extremities and reported it as “disseminated eruptive type”. Garcia-Gavin et al. [50] defined the eruptive variant as having more than 30 lesions. Their case was unique in having a rapid onset within less than a month. Some of their cases regressed, leaving hyperpigmentation, which is interesting since CCA is typically hypo- or amelanotic compared to the adjacent healthy skin and allows for the speculation that some of these lesions may have been pigmented.

#### 3.1.8. Cystic CCA

Delacretaz et al. [5], in the 1960s, described marked follicular thickening in one case of CCA. However, the involvement of the follicular cyst epithelium was first reported by Hamaguchi and Penneys [51]. Changes in CCA were noted both in the epidermis and follicular epithelium, which showed a trichilemmal pattern of keratinization. Hair shafts were seen in the attenuated cyst cavity. They posited that the epidermal portion of CCA might have blocked the follicular ostium with secondary cyst formation and extension into the infundibulum. There are no other reports of cystic CCA.

#### 3.1.9. Linear CCA

This rare variant was noted in a 4-year-old black male on the left flank as a hypopigmented linear plaque composed of small coalescing papules [6]. The plaque resembled epidermal nevus and lichen striatus, which are lesions distributed along Blaschko’s lines. Histological findings were typical of CCA.

#### 3.1.10. Subungual CCA

Cheng et al. [52] reported an interesting and only documented case of subungual papule with longitudinal splitting of the right third fingernail of an elderly male. There was no history of trauma, psoriasis or any other inflammatory condition. Histological examination showed markedly irregular and endophytic acanthosis of clear cell-containing squamous epithelium with neutrophil and lymphocyte exocytosis. PAS preparation showed abundant diastase-labile staining of keratinocytes. No recurrence was noted after surgical removal in the following six months, and the nail plate grew back with normal appearance.

#### 3.1.11. Trichilemmal CCA

The lesion described in our first case report demonstrates features of CCA including psoriasiform acanthosis, pale keratinocytes, PAS positivity, a diastase-labile staining pattern and sharp demarcation from the adjacent uninvolved epidermis. However, it differs from CCA in having no neutrophil exocytosis. Furthermore, in addition to pale cytoplasm, large clear perinuclear vacuoles were noted, reminiscent of outer sheath keratinocytes and keratinocytes of trichilemmoma. Peripheral palisading of basal keratinocytes was also identified.

One may argue that this case is merely a trichilemmoma which was also incidentally first described in 1962. Keratinocytes in trichilemmoma similarly contain cytoplasmic glycogen, and hence the demarcation between lesional and normal epidermis is also noted. The clinical and histological findings in our case, however, are not those of an exo-endophytic folliculocentric lesion located on the face. This lesion is a horizontally oriented papule on the neck, unlike trichilemmoma, which is vertically oriented. Thick eosinophilic basement membrane typical of trichilemmoma is also not seen in our case. Sharp demarcation accentuated by PAS staining in our case is tinctorial, similar to that seen in CCA. The demarcation in trichilemmoma is both tinctorial and architectural in the form of epithelial collarettes. We considered calling this lesion a “trichilemmal acanthoma”, however, due to the findings mentioned above, we prefer to document this case as a trichilemmal variant of CCA.

In the discussion of CCA versus trichilemmoma, an argument regarding the nomenclature of CCA is brought to light which is also reflected in the literature, which is whether to call these lesions “pale cell” or “clear cell” acanthoma. Keratinocyte cytoplasm in trichilemmoma is typically clear, while it is pale in keratinocytes of CCA. Perhaps the more appropriate term for CCA is pale cell acanthoma; however, this is not the objective of this paper. Another interesting fact is that CCA and GA, like trichilemmomas, are also reported to be associated with Cowden syndrome. Since all three of these lesions show cytoplasmic glycogen accumulation, there may be a disturbance of glycogen metabolisms in keratinocytes in patients with Cowden syndrome.

Based on immunohistochemical findings, Hashimoto et al. [53] have suggested the outer root sheath derivation of CCA. However, this has not been the predominant thought of other authors. Zedek et al. [54] reviewed fourteen CCAs, ten trichilemmomas and seven cases of psoriasis using conventional microscopy, PAS and immunohistochemistry and found that, immunohistochemically, CCA resembled psoriasis and not trichilemmoma. In our case, the CD34 stain was entirely negative while brightly highlighting the adjacent papillary vessels.

**Figure 9:**
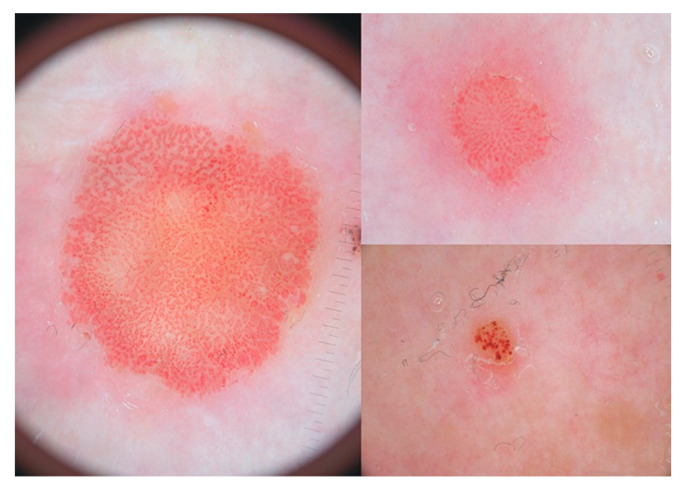
Dermoscopic examination reveals dotted vessels arranged as strings of pearls. Reprinted with permission from Tiodorovic-Zivkovic et al. [55].

Dermoscopic examination in our case was not performed. Dermoscopy in CCA reveals a “string of pearls” appearance, dotted vessels arranged linearly in circular pattern [55] (Figure 9). This appearance, however, is not characteristic of CCA and can be seen in Bowen’s disease [56].

## 4. Conclusions

In this paper, we have described a novel variant of CCA and added one more example of the rare polypoid variant. The novel trichilemmal variant may pose a diagnostic quandary to histopathologists since it shows a silhouette compatible with CCA but exhibits cytologic features suggestive of trichilemmoma. Negative CD34 staining will readily distinguish the two conditions.

We also expand the list of variants of CCA so that the clinicians are aware of its broader spectrum of clinical presentations. As more variants of these cases are reported, the list of variants could be expanded. Furthermore, the research could be targeted towards the underlying etiology of CCA, as well as early diagnosis in relation to the various clinical presentations. CCA may be suspected in patients presenting with verrucous, cystic, pigmented and subungual lesions and in lesions that follow Blaschko’s lines. Histopathologists must also be aware of the expanding subtypes of CCA. It would be helpful to keep CCA in the differential during the microscopic examination of verrucous, pigmented, polypoid or cystic squamoproliferative lesions.

Furthermore, since trichilemmoma, CCA and GA are all reported to be associated with Cowden syndrome and show cytoplasmic glycogen accumulation in keratinocytes, a possibility of disordered glycogen metabolism in keratinocytes in patients with Cowden syndrome is conceivable. Further research to substantiate this claim is, however, warranted.

## Figures and Tables

**Table 1:** List of clinical and histologic types of Clear Cell Acanthoma.

Type	First Described	Author	Age	Gender	Location of CCA
Conventional	1962	Degos and Civatte	5–7th decade	No specific inclination	Distal lower extremity
Giant	1966	Duperrat et al.	Unknown	Unknown	Leg
Mucosal	1974	Weitzner	63-year-old	Male	Vermilion mucosa of the lower lip
Verrucous	1987	Toni Alvarez	72-year-old	Female	Unknown
Polypoid	1990	Petzelbauer et al.	28-year-old	Female	Neck
Atypical	1991	Grunwald et al.	7th decade	Male:Female equally	Forehead
Pigmented	1994	Sanchez and Iglesias	60-year-old	Male	Abdominal region
Eruptive	1994	Burg et al.	38-year-old	Female	Multiple lesions on legs, arms, trunk
Cystic	1995	Hamaguchi et al.	Middle aged	Male	Suprapubic region
Linear	2012	Tempark and Shwayder	4-year-old	Male	Flank
Subungual	2014	Chun-Yu Cheng et al.	70-year-old	Male	Fingernail bed
Trichilemmal	2020	Usmani and Qasim	74-year-old	Female	Neck
